# Surface plasmon-driven photoelectrochemical water splitting of a Ag/TiO_2_ nanoplate photoanode†

**DOI:** 10.1039/d1ra09070d

**Published:** 2022-01-20

**Authors:** Piangjai Peerakiatkhajohn, Jung-Ho Yun, Teera Butburee, Waraporn Nisspa, Supphasin Thaweesak

**Affiliations:** Faculty of Environment and Resource Studies, Mahidol University Nakhon Pathom 73170 Thailand piangjai.pee@mahidol.ac.th; Nanomaterials Centre, School of Chemical Engineering and Australian Institute for Bioengineering and Nanotechnology (AIBN), The University of Queensland St Lucia QLD 4123 Australia j.yun1@uq.edu.au; National Nanotechnology Center, National Science and Technology Development Agency 111 Thailand Science Park Pathum Thani 12120 Thailand teera.but@nanotec.or.th; Division of Science and Technology, Faculty of Science and Technology, Phetchaburi Rajabhat University Phetchaburi 76000 Thailand waraporn.bun@mail.pbru.ac.th; Department of Chemical Engineering, Faculty of Engineering, Burapha University Chon Buri 20131 Thailand supphasin@eng.buu.ac.th

## Abstract

A silver/titanium dioxide nanoplate (Ag/TiO_2_ NP) photoelectrode was designed and fabricated from vertically aligned TiO_2_ nanoplates (NP) decorated with silver nanoparticles (NPs) through a simple hydrothermal synthesis and electrodeposition route. The electrodeposition times of Ag NPs on the TiO_2_ NP were crucial for surface plasmon-driven photoelectrochemical (PEC) water splitting performance. The Ag/TiO_2_ NP at the optimal deposition time of 5 min with a Ag element content of 0.53 wt% demonstrated a remarkably high photocurrent density of 0.35 mA cm^−2^ at 1.23 V *vs.* RHE under AM 1.5G illumination, which was 5 fold higher than that of the pristine TiO_2_ NP. It was clear that the enhanced light absorption properties and PEC performance for Ag/TiO_2_ NP could be effectively adjusted by simply controlling the loading amounts of metallic Ag NPs (average size of 10–30 nm) at different electrodeposition times. The superior PEC performance of the Ag/TiO_2_ NP photoanode was attributed to the synergistic effects of the plasmonic Ag NPs and the TiO_2_ nanoplate. Interestingly, the plasmonic effect of Ag NPs not only increased the visible-light response (*λ*_max_ = 570 nm) of TiO_2_ but also provided hot electrons to promote photocurrent generation and suppress charge recombination. Importantly, this study offers a potentially efficient strategy for the design and fabrication of a new type of TiO_2_ hybrid nanostructure with a plasmonic enhancement for PEC water splitting.

## Abstract 2

A hybrid nanostructure Ag/TiO_2_ photoelectrode for PEC water splitting with a remarkable high photocurrent density, 0.35 mA cm^−2^ (5 fold higher than that of the pristine TiO_2_ photoeletrode) was fabricated by a facile one-pot hydrothermal and electrodeposition method.

## Graphical abstract

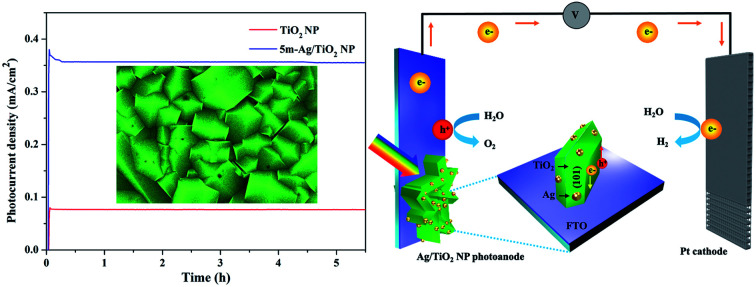

## Introduction

Photoelectrochemical water splitting is a promising approach to produce renewable hydrogen fuel from abundant solar energy. Typically, a photoelectrode should provide strong optical absorption, good stability, effective carrier separation, and less recombination of electron–hole pairs in order to meet the requirements of an efficient PEC system.^1–3^ Since its inception in 1972 by Fujishima and Honda^4^ many researchers have focused on TiO_2_ due to its relatively high reactivity, chemical stability, low cost, and environmentally friendly features.^5–7^ Nevertheless, TiO_2_ has several drawbacks such as its relatively wide band gap (∼3.2 eV for anatase), making it active only in the ultraviolet (UV) light region, which constitutes only about 5–7% of the solar spectrum.^8–10^ In addition, TiO_2_ has a high recombination rate of photoexcited electron–hole pairs, leading to a lower PEC performance.^5^ Thus, improving visible-light response and suppressing charge recombination in TiO_2_ are great challenges for efficient hydrogen production *via* PEC water splitting.^3,11^ Many strategies have been demonstrated to overcome these drawbacks, such as metal and non-metal doping,^12–15^ coupling with other semiconductors,^16–19^ and noble metal deposition.^20–22^

In particular, engineering of nanostructures such as one-dimensional (1D),^23–25^ two-dimensional (2D),^26–28^ and three-dimensional (3D)^29–31^ structures is an approach to promote effective charge separation; especially, the 2D TiO_2_ structure is an ideal building block for further nanoengineering of heterostructure materials for enhanced PEC water splitting application. Additionally, the noble metal deposition has proved to be an effective technique to extend the photoresponse of TiO_2_ to the visible light range and suppress the recombination of the photogenerated electrons–holes. Among the noble metals, silver (Ag) is an attractive metal due to its strong surface plasmon resonance (SPR) effect along with extraordinary physicochemical properties and less expensive compared to gold, palladium, and platinum.^32,33^ Various studies of Ag–TiO_2_ composites for efficient PEC water splitting have been demonstrated;^34–39^ for instance, Peng *et al.* successfully decorated the plasmonic Ag NPs on TiO_2_ nanowires for the enhancement of PEC water splitting performance,^32^ and Hou *et al.* reported TiO_2_ nanotube arrays coupled with Ag NPs, exhibiting impressive PEC water splitting activities.^40^ A comparative table summary of recent studies for Ag/TiO_2_ photoanodes is shown in ESI Table S1.† However, there are still some concerns, such as low PEC performance, complicated synthesis procedures, and complex structural fabrication.

In this work, an environmentally benign, economical, and facile technique to synthesize Ag-deposited TiO_2_ nanoplates (Ag/TiO_2_ NP) as a photoanode was reported, by the combination of a hydrothermal method and an electrodeposition technique. The Ag NP deposition can be achieved by several methods such as electrodeposition,^34,37,41^ photodeposition,^35,36,42^ and chemical reduction.^43–45^ Among various techniques, electrodeposition has arisen as a promising technique for fabricating photoelectrodes due to its relative simplicity, low cost, and good dispersion, and improves interfacial deposition between the deposited layer and supporting electrode substrate. Furthermore, the most attractive feature of electrodeposition is its feasibility in terms of particle coverage density and properties related to the catalytic activity can be easily controlled by tuning the electrolyte composition, deposition time, and applied potential when compared to other Ag NP fabrication techniques.^37,46^

The Ag/TiO_2_ NP photoanodes were also characterized using various spectroscopic, microscopic, crystallographic, and photoelectrochemical techniques to determine the optical, physico-chemical, and electrochemical properties. Therefore, the transfer-enhancement synergistic mechanism is proposed to understand the role of the plasmonic effect of Ag NPs.

## Results and discussion

**Fig. 1:**
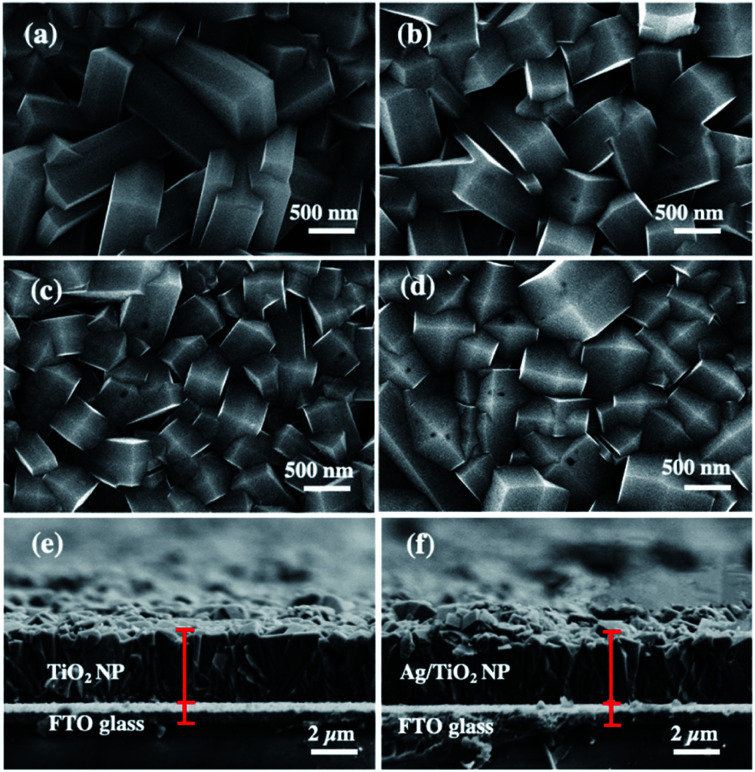
FE-SEM images of (a) TiO_2_ NP, (b) 1m-Ag/TiO_2_, (c) 3m-Ag/TiO_2_, and (d) 5m-Ag/TiO_2_ NP photoanodes, and cross-section SEM images of (e) TiO_2_ NP and (f) 5m-Ag/TiO_2_ NP photoanodes.

Fig. 1(a)–(d) are the SEM images of the structural morphology from the top view and Fig. 1(e) and (f) are the cross-sectional view of the as-prepared TiO_2_ and Ag/TiO_2_ photoanodes. Fig. 1(a) shows the top view SEM image of the TiO_2_ layer which confirms the formation of dense vertically aligned 2D nanoplates with exposed {101} and {001} facets.^47,48^ According to our previous study,^47^ it was found that protons (H^+^) and fluoride ions (F^−^) play a synergistic role in controlling the morphology and crystal phases of TiO_2_. The ion of F^−^ changes the crystal phases of TiO_2_ from rutile to anatase with low-index facets, while increasing amount of H^+^ promotes the growth of the {001} facet.^49^ The percentage of the {001} facet synthesized in this work was quantified by Raman spectroscopy, which was 9.97% (ESI Fig. S1†).

Fig. 1(b)–(d) show the circular patches of the Ag deposited onto the TiO_2_ NP. Cross-sectional images in Fig. 1(e) and (f) display the film thicknesses of TiO_2_ and 5m-Ag/TiO_2_ NP photoanodes, which are approximately 3 μm.

**Fig. 2:**
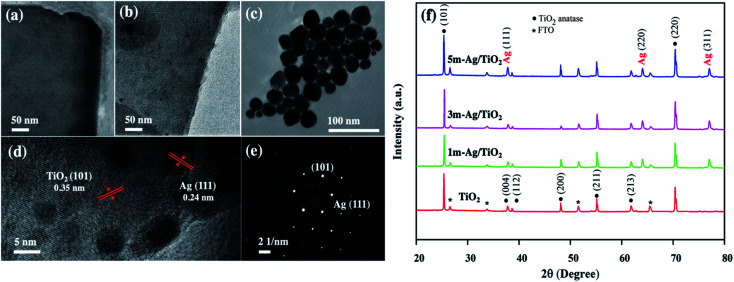
TEM images of (a) TiO_2_ NP, (b) Ag/TiO_2_ NP, and (c) Ag NPs, (d) HRTEM image of Ag/TiO_2_, (e) SAED pattern of Ag/TiO_2_, and (f) XRD patterns of the photoanode.

The TiO_2_ and Ag/TiO_2_ photoanodes were analyzed by transmission electron microscopy (TEM) and high-resolution transmission electron microscopy (HRTEM). The TEM images of TiO_2_ and 5m-Ag/TiO_2_ NP photoanodes as shown in Fig. 2(a) and (b) reveal the nanoplate structure of TiO_2_ and distribution of Ag NPs on the TiO_2_ structure with the diameters of about 10 to 30 nm, respectively. Fig. 2(c) displays the TEM image of spherical shape Ag NPs with an average size range of 10–30 nm for the electrodeposition process. Furthermore, Fig. 2(d) presents the HRTEM image of the Ag/TiO_2_ NP photoanode which confirms the coexistence of TiO_2_ and Ag NPs with a clear lattice fringe spacing of 0.35 nm and 0.24 nm, in accordance with the *d*-spacings of (101) of anatase TiO_2_ and (111) of Ag NPs, respectively.^41,50^ Additionally, Fig. 2(e) shows a sharp selected area electron diffraction (SAED) pattern, further confirming that the Ag/TiO_2_ NP is a single crystalline anatase structure with Ag NPs (111). Fig. 2(f) illustrates the XRD patterns of the synthesized TiO_2_ NP and Ag/TiO_2_ photoanodes. TiO_2_ NPs exhibit the major peaks at 2*θ* values of 25.28°, 37.8°, 38.58°, 48.05°, 55.06°, 62.12°, and 70.03°, which correspond to the characteristic peaks of anatase at (101), (004), (112), (200), (211), (213) and (220), respectively (JCPDS no. 21-1272). The Ag/TiO_2_ NP photoanode exhibits the peaks of Ag nanoparticles deposited on the TiO_2_ NP surface at 2*θ* of 38.12°, 64.42°, and 77.47°, corresponding to (111), (220) and (311) planes of Ag, respectively (JCPDS no. 04-0783). This result can indicate the existence of crystalline Ag nanoparticles in the nanocomposites of Ag/TiO_2_ NP.

**Fig. 3:**
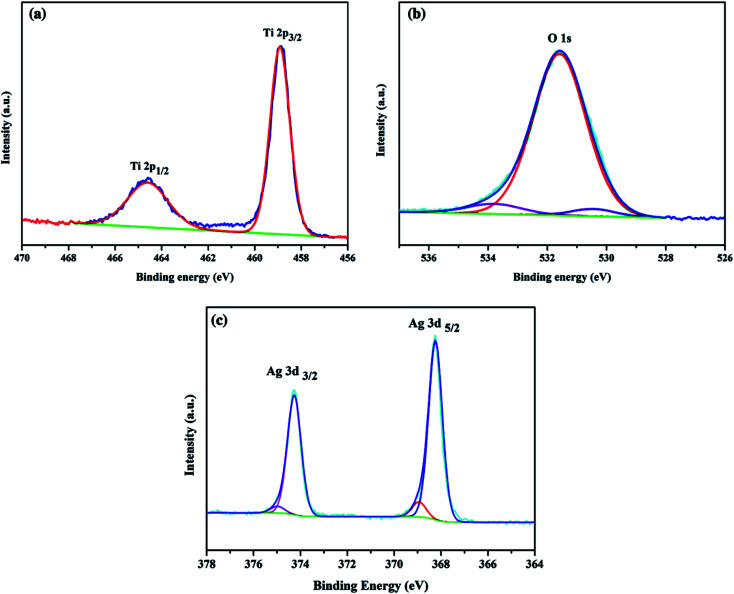
XPS spectra of (a) Ti 2p, (b) O 1s and (c) Ag 3d samples of the Ag/TiO_2_ photoanode.

The elemental composition and chemical state of the Ag/TiO_2_ photoanode were investigated by high-resolution XPS spectra as shown in Fig. 3. Fig. 3(a) illustrates the XPS spectra of Ti 2p; there are two major peaks with binding energies at 464.9 eV and 459.1 eV, corresponding to Ti 2p_3/2_ and Ti 2p_1/2_, respectively. Fig. 3(b) exhibits the O 1s region, and the peak at 530.0 eV can be assigned to oxygen atoms in the TiO_2_ lattice.^51^Fig. 3(c) shows two peaks at binding energies of 367.9 eV (Ag 3d_5/2_) and 373.9 eV (Ag 3d_3/2_), which were close to those expected for metallic Ag^0^ (368.0 eV and 374.0 eV).^9,35,52^ Thus, Ag NPs on TiO_2_ NP were mainly in the metallic state, which is consistent with the HRTEM and XRD results.

**Fig. 4:**
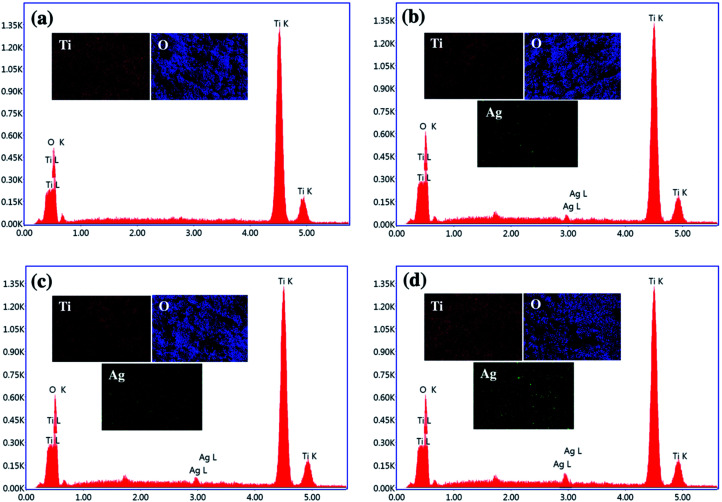
The EDS spectra and elemental mapping images of (a) TiO_2_ NP, (b) 1m-Ag/TiO_2_, (c) 3m-Ag/TiO_2_, and (d) 5m-Ag/TiO_2_ NP photoanodes.

In addition, the existence of Ag NPs on TiO_2_ was confirmed by energy-dispersive X-ray spectroscopy (EDS) as shown in Fig. 4(a)–(d). The EDS results reveal that the TiO_2_ NP contained peaks of Ti and O elements, while Ag/TiO_2_ NP contained peaks of Ti and O, and an additional peak of Ag at ∼2.9 eV, indicating that the Ag NPs in various Ag/TiO_2_ NP samples are mainly in the metallic form. In addition, elemental mapping depicts the uniform distribution of Ti, O and Ag atoms in the Ag/TiO_2_ NP. The weight percentages of Ag element in Ag/TiO_2_ NP of different electrodeposition times at 1, 3, and 5 min were 0.12, 0.34, and 0.53 wt%, respectively. The EDS results indicate that the amount of Ag nanoparticles increases with the extension of electrodeposition time which is in good agreement with the XRD results.

**Fig. 5:**
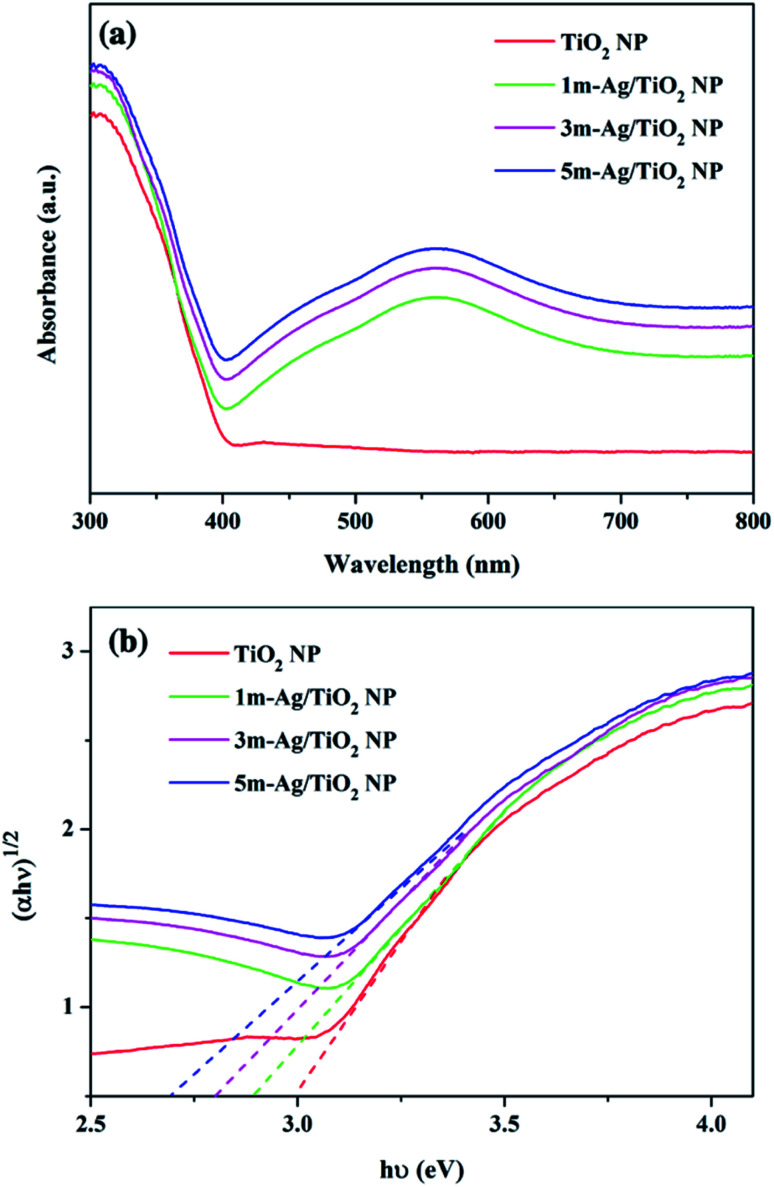
(a) UV-Vis absorption spectra and (b) Tauc plot of the TiO_2_ and Ag/TiO_2_ NP photoanodes.

UV-Vis absorption spectra of the pristine TiO_2_ and Ag/TiO_2_ NP photoanodes are shown in Fig. 5(a). The pristine TiO_2_ and Ag/TiO_2_ NP photoanodes display similar absorption intensity in the UV region. The absorption edge around 380 nm of the pristine TiO_2_ NPs is ascribed to the band-to-band transition of anatase TiO_2_.^53^ The absorption spectrum of Ag/TiO_2_ NP photoanodes shows a broad absorption band in the range of 400–700 nm with the maximum absorption at 570 nm compared to that of the bare TiO_2_.

The peak broadening is observed towards a higher wavelength which could be attributed to the SPR effect which was induced by the spatial confinement of electrons in Ag^0^ particles on the surface of TiO_2_ NP and the high dispersion of Ag on the TiO_2_.^54,55^ Furthermore, the Tauc equation was applied to calculate the band gap energy (*E*_g_) as follows:(*αhν*)^*n*^ = *A*(*hν* − *E*_g_)where *α* is the absorption coefficient, *h* is Planck's constant (4.136 × 10^−15^ eV s), ν is the light frequency (s^−1^) and *E*_g_ is the optical band gap. In this study, the energy band gap of the photoelectrodes was calculated with the direct band gap *n* = 1/2. Hence, the band gap of the TiO_2_ NP photoanode is around 3.12 eV, while those of 1m-Ag/TiO_2_ NP, 3m-Ag/TiO_2_ NP, and 5m-Ag/TiO_2_ NP photoanodes are 2.89, 2.79, and 2.68 eV, respectively, as shown in Fig. 5(b). A similar characteristic of shifted absorption edges and decreased energy bandgap of TiO_2_ modified with Ag NPs agreed with other studies.^32,56^ The visible light absorption broadening and narrowing energy bandgap may contribute to the PEC performance enhancement of the Ag/TiO_2_ NP photoanodes.

**Fig. 6:**
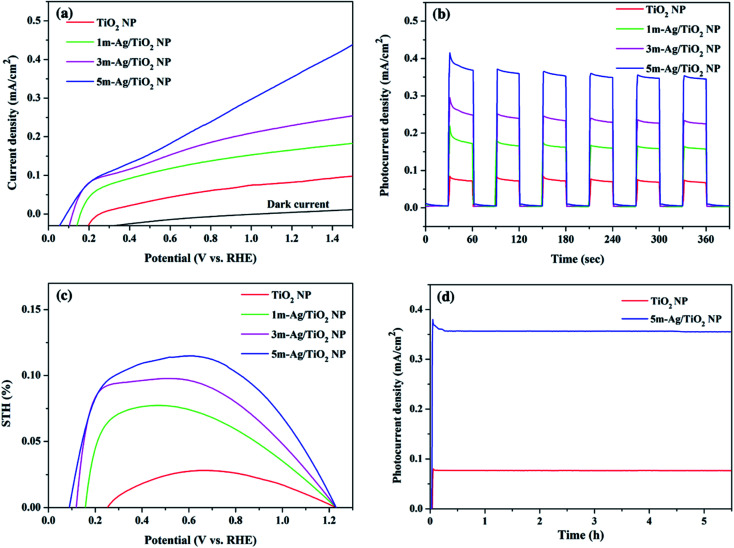
(a) The linear sweep voltammetric (*I*–*V*) curves, (b) transient photocurrent response (*I*–*t*) of TiO_2_ and Ag/TiO_2_ NP photoanodes at 1.23 V (*vs.* RHE) under simulated AM 1.5G illumination, (c) STH efficiency based on the *I*–*V* curve of TiO_2_ and 5m-Ag/TiO_2_ NP photoanodes, and (d) long-term stability test of TiO_2_ and Ag/TiO_2_ NP photoanodes at 1.23 V (*vs.* RHE) under simulated AM 1.5G illumination (100 mW cm^−2^).

The linear sweep voltammetric (*I*–*V*) curves for the pristine TiO_2_ and the Ag/TiO_2_ NP photoanodes were measured in a potential range from 0 to 1.5 V *vs.* RHE in 0.5 M Na_2_SO_4_ electrolyte under simulated AM 1.5G illumination as shown in Fig. 6(a). The photo generated potential of Ag/TiO_2_ photoanodes immediately shifts to a more negative value due to the sudden generation of photogenerated electron–hole pairs under light illumination. Obviously, the shift of the onset potential toward the lower bias when more Ag NPs were deposited is due to the lower band bending and the better charge transport.^57,58^ The photocurrent density of all Ag/TiO_2_ NP photoelectrodes is higher than that of TiO_2_ NP and increases significantly at all potentials. This could be attributed to a better electron–hole separation and SPR effect of Ag NPs, which is consistent with the light absorption spectra and the narrowing energy bandgap. Fig. 6(b) presents the transient photocurrent response of TiO_2_ and Ag/TiO_2_ NP photoanodes with chopping light at 1.23 V (*vs.* RHE) under simulated AM 1.5G illumination. The pristine TiO_2_ NP photoanode exhibits a photocurrent density of 0.07 mA cm^−2^, comparable to other reported PEC systems by the TiO_2_ photoanode.^59,60^ Upon deposition of Ag NPs at different electrodeposition times, the photocurrent density of 1m-Ag/TiO_2_, 3m-Ag/TiO_2_, and 5m-Ag/TiO_2_ NP photoanodes is drastically increased to 0.15, 0.22, and 0.35 mA cm^−2^, respectively. Interestingly, the photocurrent density of the 5m-Ag/TiO_2_ NP photoanode is ∼5 times higher than that of the pristine TiO_2_ NP photoanode. For photoanodes with longer Ag NP deposition time than 5m-Ag/TiO_2_ NP photoanodes, 7m-Ag/TiO_2_ NP and 10m-Ag/TiO_2_ NP, the photocurrent density decreases slightly to 0.30 and 0.27 mA cm^−2^, respectively with a positive shift of onset potential as shown in ESI Fig. S2.† The excess amount of Ag NPs on the TiO_2_ NP photoanode function as recombination sites, thus decreasing the photoelectrochemical activity. This phenomenon is also reported by other studies.^37,39^Fig. 6(c) shows the calculated STH efficiencies of these photoelectrodes based on the *I*–*V* characteristics. The 5m-Ag/TiO_2_ NP photoanode exhibits the maximum STH value of approximately 0.12%. Additionally, the 5m-Ag/TiO_2_ NP photoelectrode shows extraordinarily high stability for a stable photocurrent for at least 5 hours, as shown in Fig. 6(d). This result indicated that surface modification of TiO_2_ with an economical noble metal (Ag) is an effective strategy to improve the PEC performance which is comparable to other studies (ESI Table S1†).

**Fig. 7:**
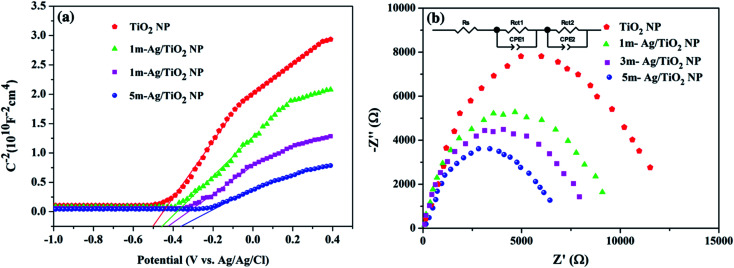
(a) Mott–Schottky curves and (b) Nyquist plots of the electrochemical impedance spectroscopy (EIS) measurements of various photoelectrodes. The inset gives the equivalent model circuit used to fit the impedance data.

To investigate the role of deposited Ag NPs in the enhancement of photoelectrochemical performance and electronic properties of the TiO_2_ photoelectrode, the Mott–Schottky (MS) curves were investigated as shown in Fig. 7(a). The slopes of the TiO_2_ and all Ag/TiO_2_ photoanodes are all positive, indicating the n-type behaviour.^58,61^ In addition, the charge carrier densities (*N*_d_) can be calculated from the slopes of MS plots using the equation as follows.^62^
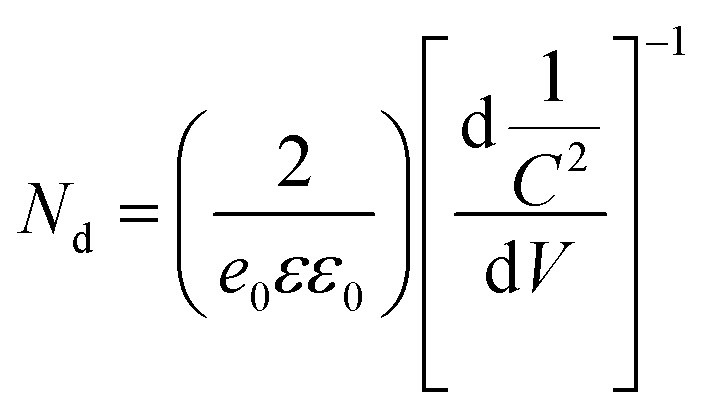
where *N*_d_ is the charge carrier density, *e*_0_ is the electron charge, *ε* is the dielectric constant of TiO_2_ (*ε* = 170), and *ε*_0_ is the permittivity of vacuum. The calculated charge carrier densities of the TiO_2_, 1m-Ag/TiO_2_, 3m-Ag/TiO_2_, and 5m-Ag/TiO_2_ were 2.393 × 10^24^ cm^−3^, 2.983 × 10^24^ cm^−3^, 4.079 × 10^24^ cm^−3^ and 6.896 × 10^24^ cm^−3^, respectively. The higher charge carrier density in all Ag/TiO_2_ photoanodes compared to that of the TiO_2_ photoanode suggested an intimate contact of Ag NPs and TiO_2_ nanoplate. Additionally, TiO_2_ shows a steeper slope than all Ag/TiO_2_ photoanodes, while the slope decreased gradually as the Ag NP deposition time increased, indicating the improvement of carrier density in Ag/TiO_2_. Furthermore, the increased electron density is responsible for the shift of the Fermi level of TiO_2_ toward the conduction band, which facilitates the charge separation.^61,63^ Moreover, a positive shift of flat band potential (*V*_fb_) of the MS plots in the presence of Ag NPs could be beneficial for the enhanced efficiency of the electron-injection. Thus, the enhancement of charge carrier density in the Ag/TiO_2_ photoanode could improve the charge transport, resulting in enhanced photocurrent density. To understand the charge transfer characteristics at the interface of the photoelectrode and electrolyte, electrochemical impedance spectra (EIS) were further recorded as shown in Fig. 7(b). The results reveal that all Ag/TiO_2_ photoanodes exhibit a smaller impedance radius compared to that of the bare TiO_2_ photoanode. The smallest impedance radius of 5m-Ag/TiO_2_ NP indicates the rapid charge transfer with lower charge transfer resistance at the electrode electrolyte interface.^61,63,64^ In addition, the equivalent model circuit (inset of Fig. 7(b)) was carried out to estimate the values of *R*_s_ representing the resistance of the system, and *R*_ct_ and CPE representing the charge transfer resistance and the capacitance, respectively (ESI Table S2†). The simulated values of charge transfer resistance follow the order TiO_2_ NP > 1m-Ag/TiO_2_ NP > 3m-Ag/TiO_2_ NP > 5m-Ag/TiO_2_ NP. The smallest value of *R*_ct_ for the 5m-Ag/TiO_2_ photoanode confirms faster charge transfer and better charge separation as compared to the bare TiO_2_ photoanode at the semiconductor electrolyte interface.^43,65^ Therefore loading Ag NPs on TiO_2_ NP further reduces the charge transfer resistance, and yields higher conductivity consequently improving the photoelectrochemical performance.

**Fig. 8:**
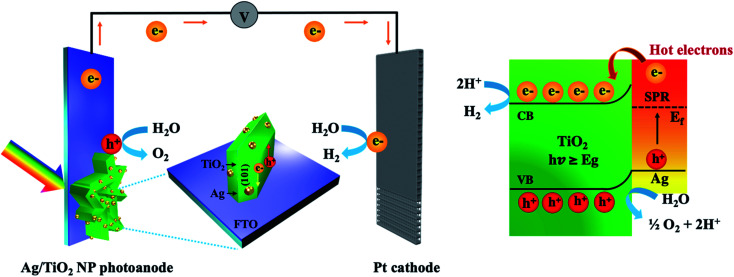
Schematic of TiO_2_ NP deposited with plasmonic Ag NPs in the PEC water splitting system.

The results of characterization and PEC measurements demonstrated that the optical property, electronic property, and photocurrent density of Ag/TiO_2_ NP photoanodes significantly depend on the electrodeposition time of Ag NP decoration. The light absorption characteristic increased gradually with the increase in deposition time, leading to the enhancement of photocurrent density. Furthermore, it could be seen that all modified TiO_2_ NP photoanodes with Ag NPs revealed higher photocurrent density than the pristine TiO_2_. Among Ag/TiO_2_ NP photoanodes, the 5m-Ag/TiO_2_ photoanode presented the highest photocurrent density of 0.35 mA cm^−2^ at 1.23 V *vs.* RHE under AM 1.5G illumination, which is ∼5 times higher compared to that of the pristine TiO_2_ NP. A proposed mechanism of the Ag/TiO_2_ NP photoanode for the enhanced PEC performance can be seen in Fig. 8. A vertically oriented TiO_2_ NP with exposed active {101}–{001} facet pairs and an interconnected framework provides an excellent architectural arrangement for facilitating charge carrier transport; for instance, Butburee *et al.* reported that the co-exposure of {101} and {010} facets could promote efficient charge separation for reduction reactions in PEC water splitting, due to their different electron affinities.^66^ There are several experiments showing that photogenerated electrons are likely to move and aggregate on the {101} facet, and *vice versa*, holes are likely to move and aggregate on the {001} facet.^67^ Besides, TiO_2_ NP arrays created more reaction sites between the photoanode and the electrolyte.^6,26^ Thus, using TiO_2_ nanostructures with good crystallinity could provide a better transfer pathway for photogenerated electrons, and assisted faster electron transport as mentioned by other studies.^68–70^ Furthermore, the deposition of Ag NPs onto TiO_2_ NP surfaces promoted significantly improved light harvesting in the broad wavelength region due to surface plasmon resonance (SPR) and effective charge transfer.^42,71,72^ In addition, HRTEM and elemental mapping exhibited that the Ag NPs with relatively uniform particle sizes distribute throughout the TiO_2_ NP. The Ag NPs play an important role for inducing more photogenerated charge carriers as an electron reservoir to suppress the charge recombination at the TiO_2_ photoanode.^13^ Under UV light illumination, the electron–hole pairs are immediately generated in TiO_2_, and the electrons in the conduction band can be directly transferred to the underneath FTO substrate for H_2_ generation at the Pt counter electrode. Under visible light irradiation, the incident light coincides with the localized surface plasmon resonances (LSPR) of the plasmonic Ag NPs and consequently the hot electrons near the Fermi level (*E*_F_) are generated and excited to the higher-energy states. These hot electrons with sufficient energy overcome the Schottky barrier formed at the interface between Ag and TiO_2_ which can significantly retard the recombination of electron–hole pairs,^54,73^ move to the conduction band of TiO_2_, and finally transfer to the Pt counter electrode *via* an external circuit for hydrogen production. And the photogenerated holes can participate in the water oxidation process or O_2_ evolution. Thus, the redox reaction and photocurrent density are improved. It is in good agreement with the study by Sang *et al.*, which described the enhanced PEC performance using Ag NPs and reduced graphene oxide (rGO) co-decorated hierarchical TiO_2_ nanoring/nanotube arrays that was mainly attributed to the effective utilization of hot electrons generated from surface plasmon resonance and effective photogenerated electron transfer of Ag NPs.^54^

## Experimental section

All chemicals were of analytical grade, and used without further purification. Titanium tetra *n*-butoxide (Ti(OC_4_H_9_)_4_), ammonium hexafluorotitanate ((NH_4_)_2_TiF_6_), and hydrochloric acid (HCl) were purchased from Sigma-Aldrich. Conductive fluorine-doped tin oxide (FTO, thickness around 2.3 mm, 15 Ω sq^−1^) glasses were used for all working electrodes (OPV Tech). All water used in the experiments was Milli-Q water (18.2 MΩ). A controlled DC power source (Agilent Technologies, Model E 3949A) supplied the required constant potential.

### Preparation of the TiO_2_ nanoplate photoanode (TiO_2_ NP)

The TiO_2_ nanoplate-like photoanode was synthesized using Ti(OC_4_H_9_)_4_ and (NH_4_)_2_TiF_6_ as precursors and HCl as a structure-directing agent in a hydrothermal reactor. The precursor solution for hydrothermal treatment was prepared by mixing 22 mL of 37% HCl in 24 mL of Milli-Q water under constant stirring at room temperature for 5 min. Then, 2 mL of Ti(OC_4_H_9_)_4_ was added into the above solution under constant stirring at room temperature for 5 min. Subsequently, 0.4 g of (NH_4_)_2_TiF_6_ was added into the mixture with continual stirring for 15 min to obtain a clear solution with pH = 5. Afterward, the as-prepared precursor was then transferred into a 50 mL Teflon-lined stainless-steel autoclave. The FTO glass substrate was cleaned by ultrasonic treatment using acetone, ethanol, and isopropanol (each for 15 min), followed by drying in a nitrogen stream and then immersed in an autoclave with the FTO side leaned against the wall. Then, the autoclave was sealed and hydrothermally treated at 170 °C for 12 h. After the hydrothermal process, the FTO substrate was brought out, rinsed with Milli-Q water, and dried in a nitrogen stream. Finally, the as-prepared TiO_2_ photoanode was subsequently annealed in air at 500 °C for 30 min with a ramping rate of 2 °C min^−1^.

### Preparation of Ag deposited TiO_2_ nanoplate photoanodes (Ag/TiO_2_ NP)

Ag NPs were synthesized by a modified Tollens' method and our previous study.^46,74^ In brief, Tollens' reagent was prepared by adding 5 mL of 0.8 M NaOH solution to 10 mL of 0.1 M silver nitrate (AgNO_3_) solution, resulting in a dark precipitate of silver oxide (AgO). Subsequently, ammonium hydroxide (NH_4_OH) solution was added drop-wise to AgO solution until a clear solution of diamminesilver(i) complex ([Ag(NH_3_)_2_]^+^) was obtained as Tollens' reagent. The formation of Ag NPs was achieved using ([Ag(NH_3_)_2_]^+^) as a Ag precursor and d-glucose (RCHO) as a reducing agent. 250 μL of Tollens' reagent solution was added to 100 mL of a 0.25 M d-glucose solution. The chemical reaction for the modified Tollens' method is as follows:

1 2AgNO_3_ + 2NaOH → Ag_2_O(s) + 2NaNO_3_ + H_2_O

2 Ag_2_O(s) + 4NH_4_OH + 2NaNO_3_ → 2[Ag(NH_3_)_2_]NO_3_ + 2NaOH

3 2[Ag(NH_3_)_2_]^+^ + RCHO + H_2_O → 2Ag^0^(s) + 4NH_3_ + RCO_2_H + 2H^+^

**Scheme 1:**
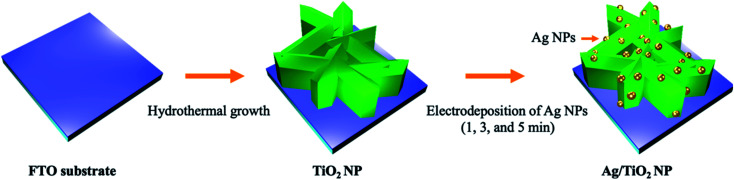
Schematic illustration of fabrication of the Ag/TiO_2_ NP photoanode.

Afterward, the solution was rapidly heated in a water bath at 70 °C under vigorous stirring until a bright yellow solution appeared. Then, the suspension solution of Ag NPs was quickly cooled down in an ice bath for the further electrodeposition process. The Ag/TiO_2_ NP photoanode was prepared by the electrodeposition method in a three-electrode system. TiO_2_ NP, Pt, and Ag/AgCl electrodes acted as the working electrode, counter electrode, and reference electrode, respectively. Electrodeposition was performed at 0.5 V *vs.* Ag/AgCl in 50 mL of the as-prepared Ag NP solution which served as the electrolyte bath. The loading content of Ag NPs over the TiO_2_ NP surface was controlled by adjusting the deposition time at 1, 3, and 5 min, which were denoted as 1m-Ag/TiO_2_, 3m-Ag/TiO_2_, and 5m-Ag/TiO_2_ NP, respectively. After electrodeposition, the deposited samples were rinsed gently with deionized water and then blow-dried by a nitrogen stream. The synthetic procedure described in the experimental section is summarized in Scheme 1.

### Characterization

The surface morphology and elemental analysis of the samples were examined using a field emission scanning electron microscope equipped with an energy-dispersive X-ray spectroscope (FE-SEM/EDX, Hitachi SU8030). TEM and HRTEM analyses were conducted using a transmission electron microscope (JEOL2100 Plus, Japan). The crystalline phases of photoanodes were characterized by X-ray diffraction (XRD; Bruker, D2 Phaser) using the Cu Kα1 radiation in a 2*θ* range of 20°–80°. The light absorption spectrum and photocatalytic activity were investigated with a UV-Vis spectrophotometer (JASCO V-630). X-ray photoelectron spectroscopy (XPS) data were measured with a Kratos Axis ULTRA X-ray photoelectron spectrometer. Furthermore, electrochemical impedance spectra were measured in an AC potential frequency range of 100 000–0.1 Hz with an amplitude of 10 mV. Z-view software was used to fit the Nyquist spectrum to obtain the equivalent circuit.

### Photoelectrochemical measurement

The photoelectrochemical measurements were performed in a standard three-electrode photoelectrochemical cell with a quartz window and tested on a CHI Instrument PSTrace 4.8. The prepared photoelectrodes, a Pt wire (1 mm diameter), and a Ag/AgCl electrode served as the working electrode, counter electrode, and reference electrode, respectively. The illumination area was set by an aperture diameter of 1 cm. An aqueous 0.5 M Na_2_SO_4_ solution was used as an electrolyte. A xenon lamp (100W, Newport LCS-100) was used to simulate sunlight and the photocurrent densities were measured under solar AM 1.5G illumination (100 mW cm^−2^). Potentials *versus* RHE were calculated using the Nernst equation *E*_RHE_ = *E*_Ag/AgCl_ + 0.0591(pH) + 0.1976 V. The solar-to-hydrogen (STH) conversion efficiencies were calculated using the values from the *I*–*V* curves under chopped light illumination with the following equation:^75^
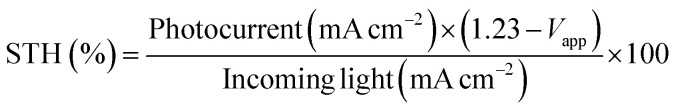


## Conclusion

In summary, we successfully synthesized TiO_2_ NP arrays by a facile one-pot hydrothermal process and decorated them with low-cost plasmonic Ag NPs by an electrodeposition. The deposition time of Ag NPs on TiO_2_ NP had a significant effect on the amount of Ag NPs on the nanocomposite for light-harvesting efficiency and PEC performance. Increasing the Ag NPs loading, the Ag/TiO_2_ NP exhibited an additional absorption band in the visible light region, indicating the narrower bandgap. Thus, photocurrent density was improved compared with the bare TiO_2_ NP photoelectrode. The PEC response of the Ag/TiO_2_ NP photoanode at a deposition time of 5 min exhibited an ∼5 times enhancement compared to the pristine TiO_2_ NP photoelectrode under AM 1.5G illumination. The higher PEC performance could be attributable to the synergistic effects of the excellent light harvesting property and hot electrons of the plasmonic Ag NPs and vertically oriented TiO_2_ NP with {101}–{001} facet pairs. Notably, this modified TiO_2_ NP with plasmonic Ag NPs is a promising photoanode for a sustainable solar energy conversion material due to simple fabrication processes, low-cost materials, high PEC performance, and excellent long-term stability.

## Author contributions

Conceptualization, P. P., J.-H. Y., T. B., and S. T.; methodology, P. P., T. B., and S. T.; validation, P. P., J.-H. Y., T. B., W. N., and S. T.; formal analysis, P. P., J.-H. Y., T. B., W. N., and S. T.; investigation, P. P. and T. B.; resources, P. P. and T. B.; visualization, P. P. and T. B.; writing – original draft preparation, P. P.; writing – review and editing, P. P., J.-H. Y., T. B., W. N., and S. T.; supervision, S. T. All authors have read and agreed to the published version of the manuscript.

## Conflicts of interest

There are no conflicts to declare.

## Supplementary Material

RA-012-D1RA09070D-s001

RA-012-D1RA09070D-s002

RA-012-D1RA09070D-s003

RA-012-D1RA09070D-s004
