# Supplementary material for: Surface plasmon-driven photoelectrochemical water splitting of a Ag/TiO2 nanoplate photoanode
Source: RSC Adv. 2022 Jan 20;12(5):2652–61. doi: 10.1039/d1ra09070d (PMC8979192; doi:10.1039/d1ra09070d)
Supplement: RA-012-D1RA09070D-s001 [file RA-012-D1RA09070D-s001.pdf]

Electronic Supplementary Material (ESI) for RSC Advances.
This journal is © The Royal Society of Chemistry 2022

Electronic Supplementary Information

Surface plasmon-driven photoelectrochemical water splitting of Ag/TiO_2 Nanoplate Photoanode

Piangjai Peerakiatkhajohn[a], Jung-Ho Yun[b], Teera Butburee[c], Waraporn Nisspa[d], and Supphasin Thaweesak [e*]

a Faculty of Environment and Resource Studies, Mahidol University, Nakhon Pathom 73170, Thailand; piangjai.pee@mahidol.ac.th

b Nanomaterials Centre, School of Chemical Engineering and Australian Institute for Bioengineering and Nanotechnology (AIBN), The University of Queensland, St Lucia, QLD 4123, Australia; j.yun1@uq.edu.au

c National Nanotechnology Center, National Science and Technology Development Agency, 111 Thailand Science Park, Pathum Thani 12120, Thailand; teera.but@nanotec.or.th

d Division of Science and Technology, Faculty of Science and Technology, Phetchaburi Rajabhat University, Phetchaburi 76000, Thailand; waraporn.bun@mail.pbru.ac.th

e Department of Chemical Engineering, Faculty of Engineering, Burapha University, Chon Buri 20131, Thailand; supphasin@eng.buu.ac.th

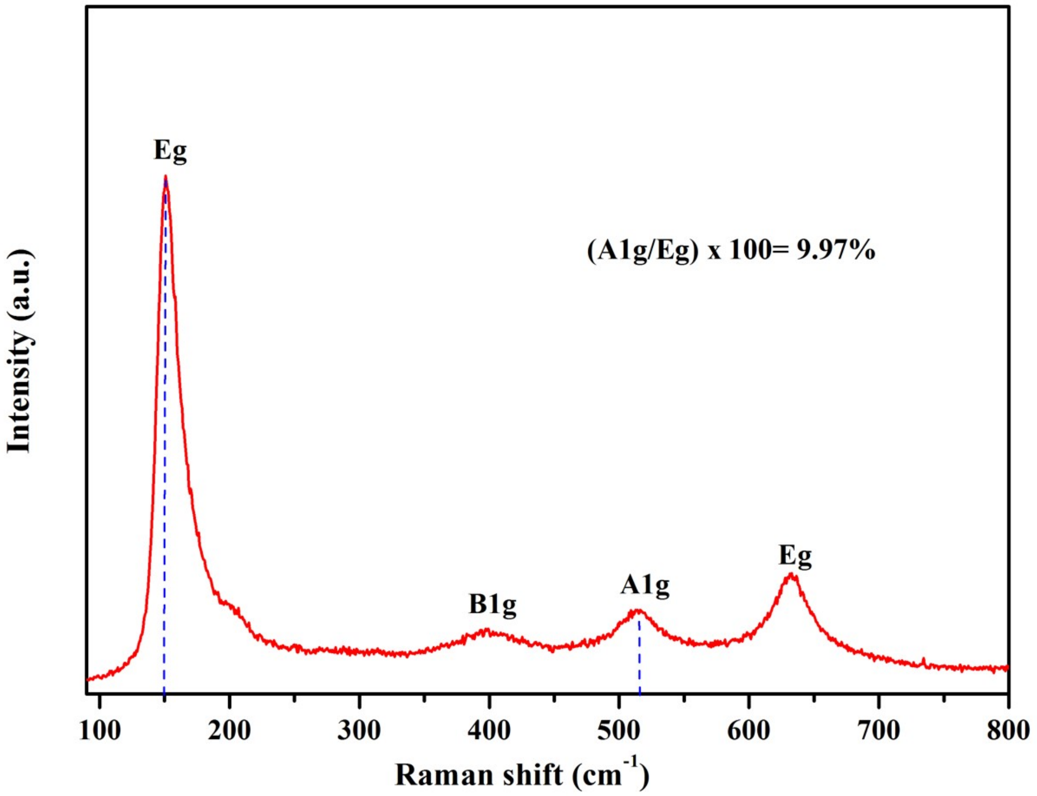

Fig. S1 Raman spectra of TiO_2 NP photoanode.

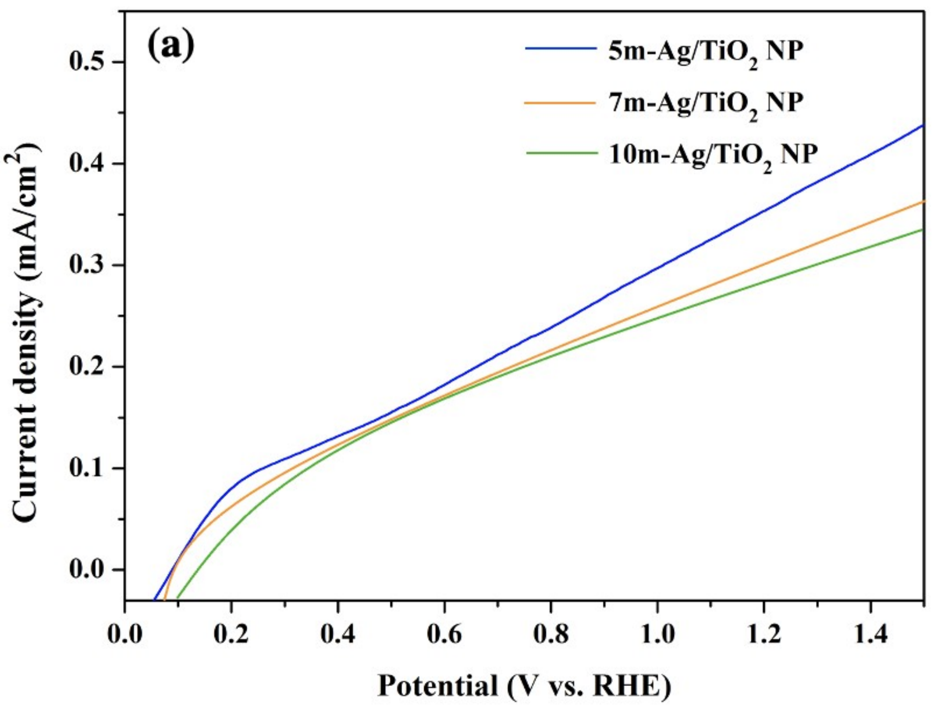

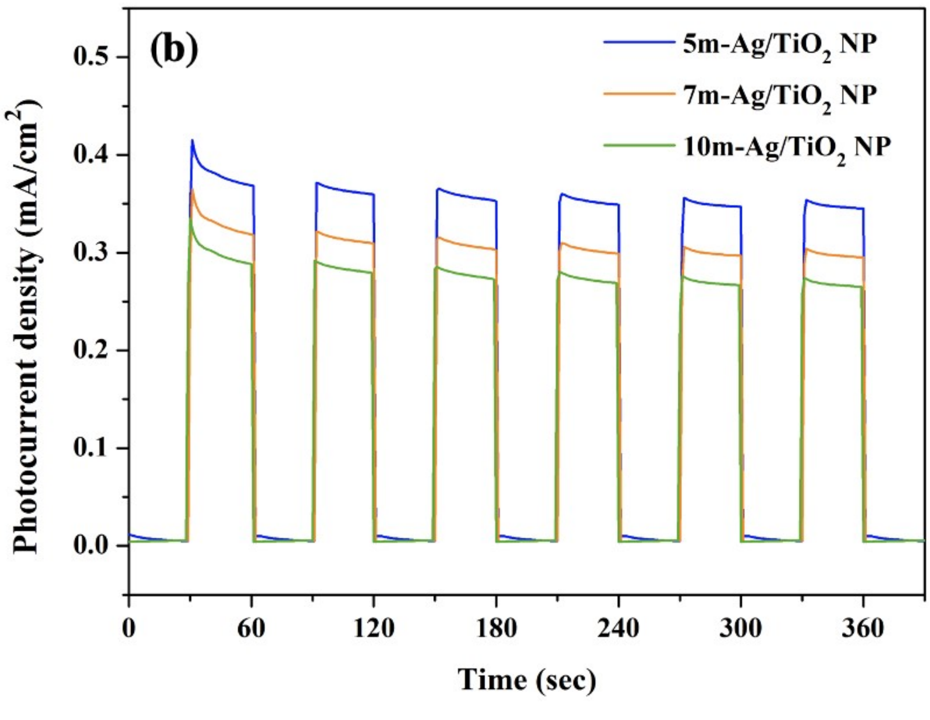

Fig. S2 The variations of photocurrent density of Ag/TiO_2 NP photanodes at different deposition time (a) linear sweep voltametric (I-V) curves and (b) Transient photocurrent response (I-t) at 1.23 V vs. RHE under simulated AM1.5G illumination.

Table S1 A summary of recent studies for Ag/TiO_2 based photoanodes in photoelectrochemical system.

Photoanode	Photocurrent density	Electrolyte/ Illumination	Method	Ref.
TiO_2 nanotube<br>Ag/TiO_2 nanotube	-<br>0.104 mA/cm^2<br>at 0.7 V vs. SCE	0.5 M Na_2SO_4,<br>300 W Xe lamp	Anodization and electrodeposition	[1]
TiO_2 nanorod<br>Ag/TiO_2 nanorod	0.014 mA/cm^2<br>0.047 mA/cm^2	0.35 M $NaSO_3$ and<br>0.25 M NaS,<br>100 mW/cm^2	Hydrothermal and photodeposition	[2]
TiO_2 nanorod<br>Ag/TiO_2 nanorod	0.012 mA/cm^2<br>0.043 mA/cm^2	0.1 M Na_2SO_4,<br>150 W xenon lamp	Hydrothermal and photodeposition	[3]
Fe/TiO_2 nanotube<br>Ag/TiO_2 nanotube	0.05 mA/cm^2<br>0.23 mA/cm^2<br>at 0.6 V vs. Ag/AgCl	0.1M Na_2S and<br>0.2 M NaOH,<br>100 mW/cm^2	Anodization and electrodeposition	[4]
TiO_2 nanotube<br>Ag/TiO_2 nanotube	0.011 mA/cm^2<br>0.1 mA/cm^2<br>at 0 V vs. Ag/AgCl	0.1M Na_2S and<br>0.2 M NaOH,<br>100 mW/cm^2	Anodization and photodeposition	[5]
TiO_2 nanoplate<br>Ag/TiO_2 nanoplate	0.07 mA/cm^2<br>0.35 mA/cm^2<br>at 1.23 V vs. RHE	0.5 M Na_2SO_4,<br>100 mW/cm^2	Hydrothermal and electrodeposition	This study

Table S2 The fitting results using the equivalent model for EIS measurements

Photoanode	R(Ω)			CPE (F)	
	**Rs**	**Rct1**	**Rct2**	**CPE1**	**CPE2**
TiO_2	7.595	113.9	66.59	4.747 $x10^{-8}$	1.579 $x10^{-7}$
1m-Ag/TiO_2	5.078	31.06	40.54	1.912 $x10^{-7}$	3.198 $x10^{-6}$
3m-Ag/TiO_2	3.522	29.74	35.93	2.349 $x10^{-7}$	3.703 $x10^{-6}$
5m-Ag/TiO_2	3.452	21.81	31.12	2.995 $x10^{-7}$	3.822 $x10^{-6}$

References

1. Z. Lian, W. Wang, S. Xiao, X. Li, Y. Cui, D. Zhang, G. Li and H. Li, Scientific Reports, 2015, 5, 10461.
2. K. Xu, Z. Liu, S. Qi, Z. Yin, S. Deng, M. Zhang and Z. Sun, RSC Advances, 2020, 10, 34702-34711.
3. K. X. Zhu Liu, Hai Yu, Zhaoqi Sun, International Journal of Energy Research, 2020, 45.
4. M. M. Momeni and P. Zeinali, Journal of Electroanalytical Chemistry, 2021, 891, 115283.
5. M. M. Momeni and P. Zeinali, Journal of Electronic Materials, 2021, 50, 5810-5818.
