# Supplementary figures and images for: Surface plasmon-driven photoelectrochemical water splitting of a Ag/TiO2 nanoplate photoanode
Source: RSC Adv. 2022 Jan 20;12(5):2652–61. doi: 10.1039/d1ra09070d (PMC8979192; doi:10.1039/d1ra09070d)

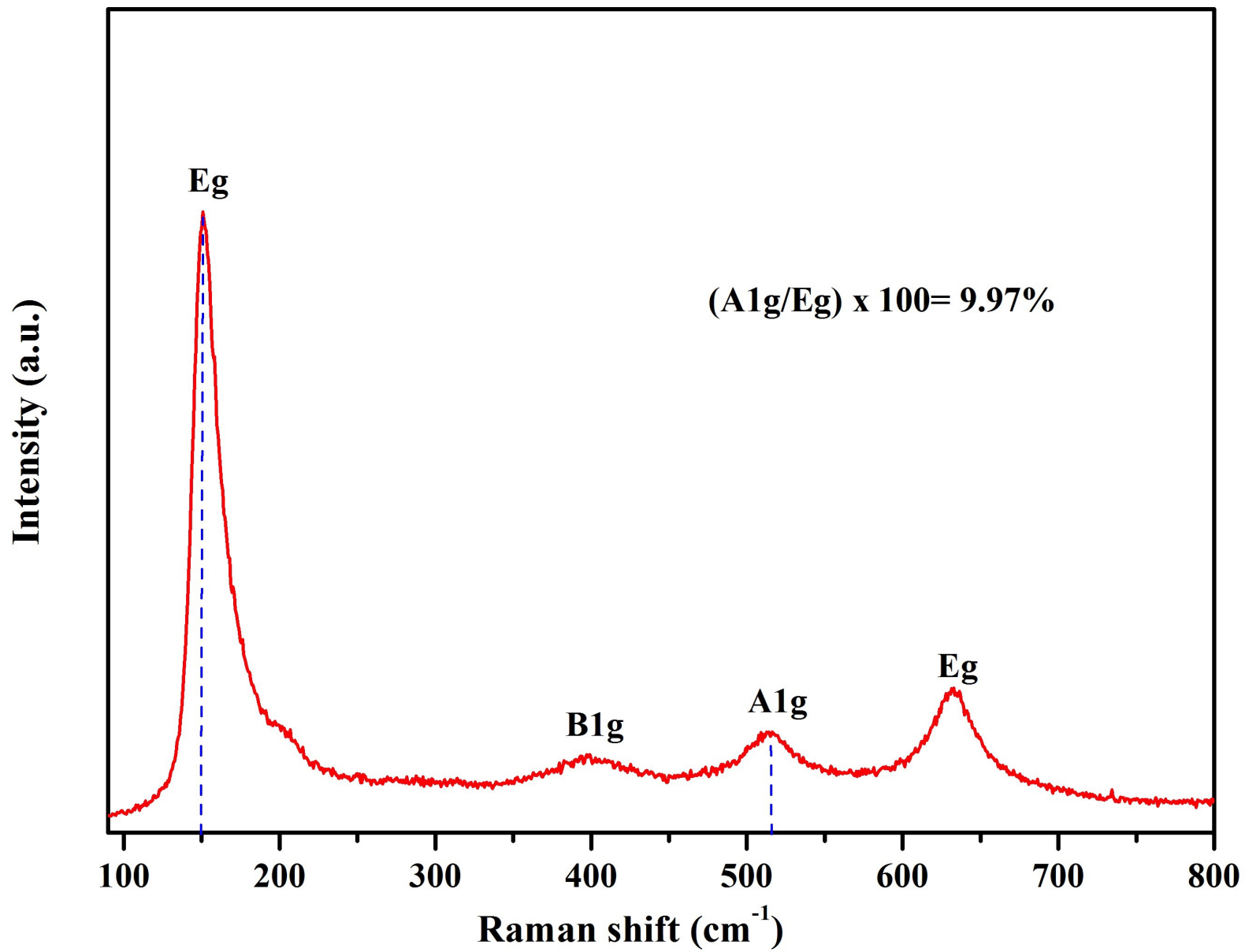

Supplement: RA-012-D1RA09070D-s003 [file RA-012-D1RA09070D-s003.pdf]

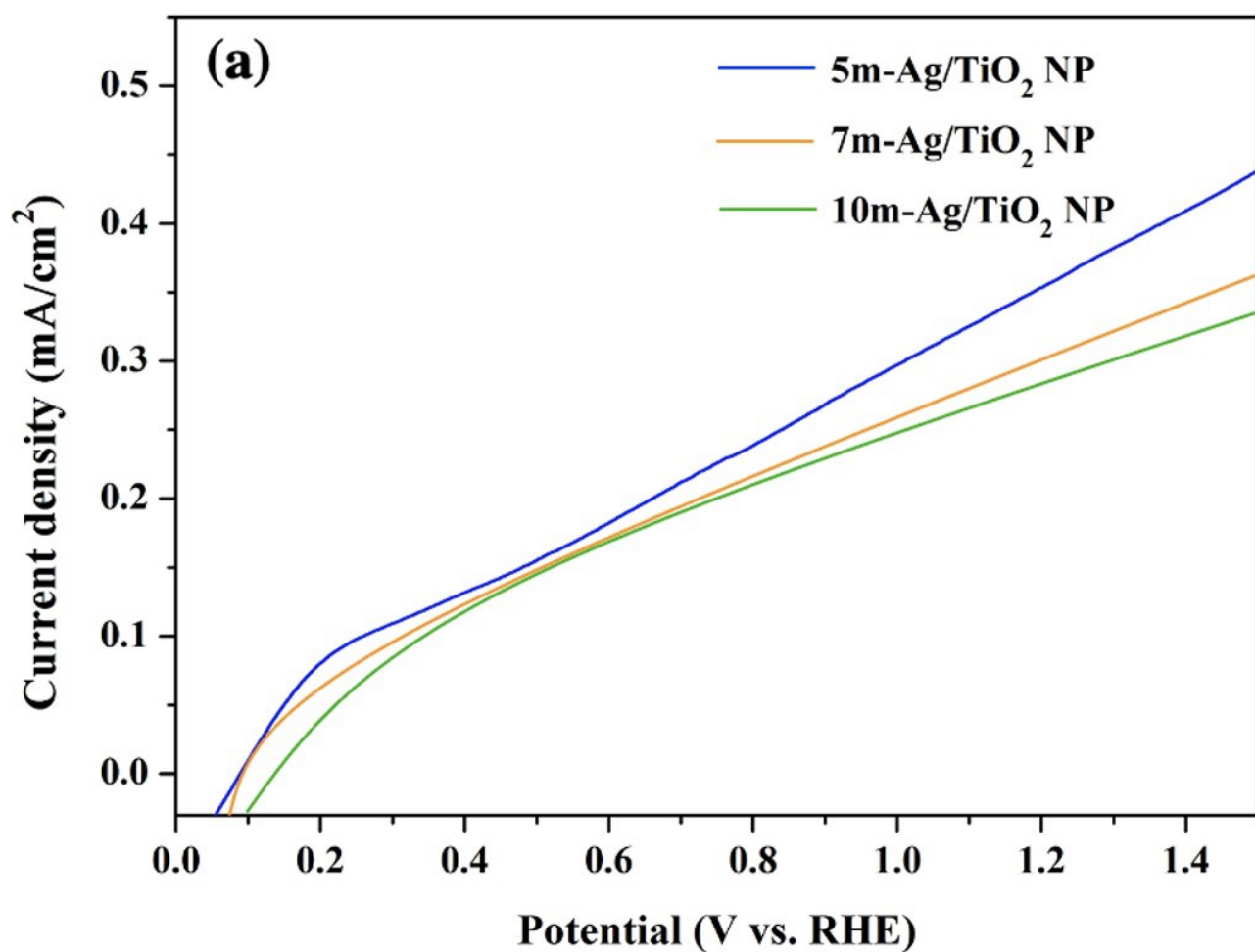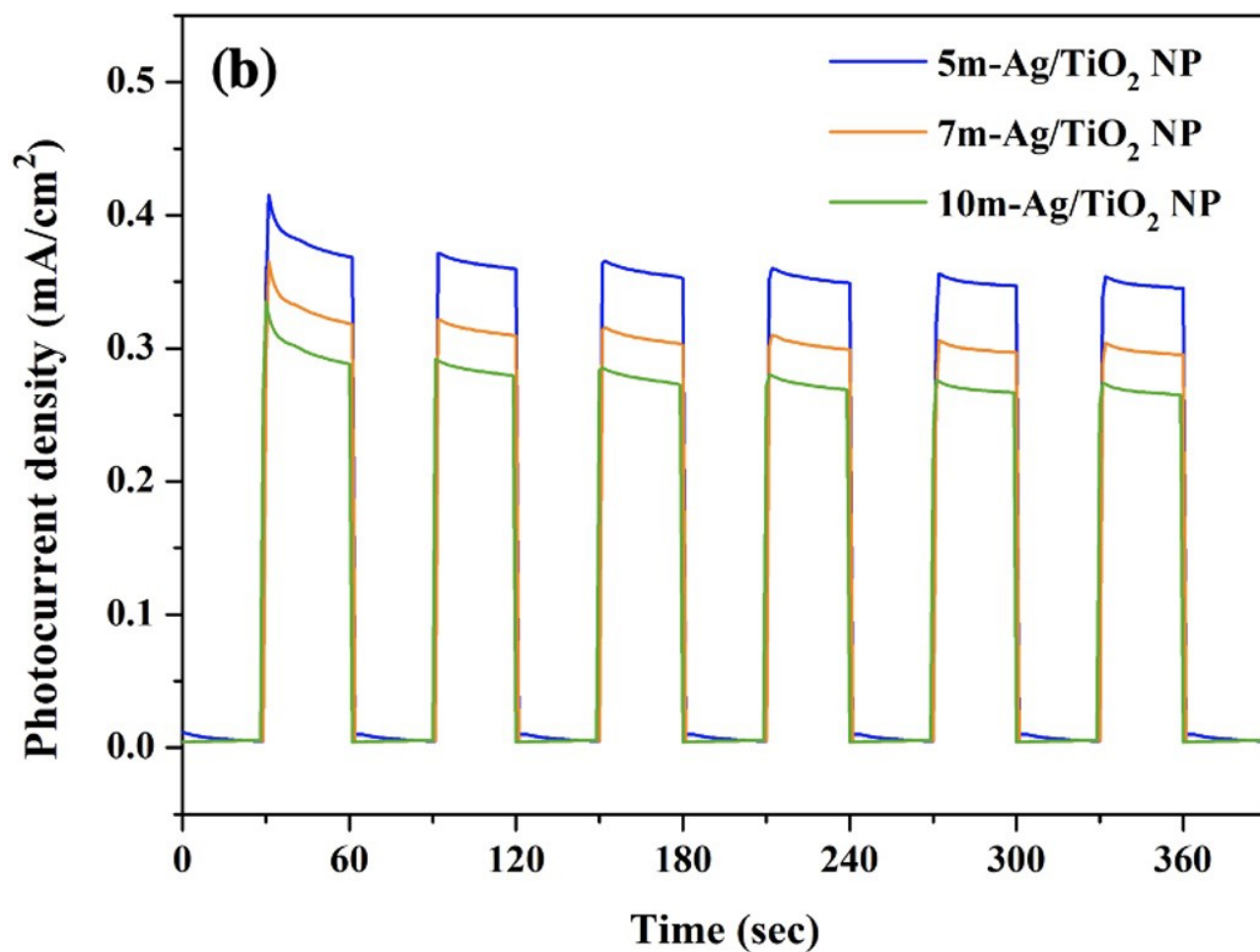

Supplement: RA-012-D1RA09070D-s004 [file RA-012-D1RA09070D-s004.pdf]
